# Thai veterinarians' perceptions of cannabidiol products for dogs with osteoarthritis: a qualitative interview study

**DOI:** 10.3389/fvets.2023.1304180

**Published:** 2023-12-14

**Authors:** Osot Nerapusee, Kumpanart Soontornvipart, Thanakrit Pettong, Nutkritta Phongsuchat, Doungkamol Lunsucheep, Chanthawat Patikorn, Sornkanok Vimolmangkang, Puree Anantachoti

**Affiliations:** ^1^Department of Social and Administrative Pharmacy, Faculty of Pharmaceutical Sciences, Chulalongkorn University, Bangkok, Thailand; ^2^Department of Veterinary Surgery, Faculty of Veterinary Science, Chulalongkorn University, Bangkok, Thailand; ^3^Department of Pharmacognosy and Pharmaceutical Botany, Faculty of Pharmaceutical Sciences, Chulalongkorn University, Bangkok, Thailand; ^4^Research Cluster for Cannabis and its Natural Substances, Chulalongkorn University, Bangkok, Thailand; ^5^Center of Excellence in Plant-Produced Pharmaceuticals, Faculty of Pharmaceutical Sciences, Chulalongkorn University, Bangkok, Thailand

**Keywords:** *Cannabis sativa*, cannabidiol, canine osteoarthritis, pain, veterinary, perception

## Abstract

**Introduction:**

The global popularity of cannabidiol (CBD) led to its approval for human use in Thailand and potential use in animals. Many studies revealed CBD's efficacy in treating chronic osteoarthritis (OA) in dogs. To facilitate tailored CBD product development for canine OA and ensure market success, this study explores Thai veterinarians' perception of CBD for canine OA.

**Methods:**

In-depth interviews were conducted with experienced veterinarians who treated OA in ≥25% of their canine patients. Interview questions covered treatment, CBD perception, and adoption. Interviews were held from January to March 2023.

**Results:**

Eleven out of twenty invited veterinarians participated in the study. Though all favored non-steroidal anti-inflammatory drugs (NSAIDs) for OA in dogs, concerns about adverse reactions, including ulcers and renal failure, emerged. CBD was widely known and was perceived positively for pain, inflammation, cancer, and epilepsy. However, half distinguished CBD from marijuana and tetrahydrocannabinol (THC). Ten expressed willingness to prescribe CBD for OA upon robust clinical evidence. Worries centered on product consistency and impurities. Many suggested CBD should be under veterinarians' supervision at first, but this can be relaxing once safety and efficacy are established. CBD products should be chewable tablets, oils, or gelatin capsules with flavors like beef, liver, pork, fish, or seafood.

**Conclusion:**

Though CBD benefits were recognized, knowledge gaps among the participants persisted, warranting robust CBD safety, efficacy, and quality evidence to ensure Thai market success. Comprehensive education, including continuing education for those in practice and incorporating CBD-related topics into the Schools of Veterinary's curriculum, is recommended.

## Introduction

Canine osteoarthritis (OA) often affects larger and older dogs (1), impacting about 20% of dogs (2, 3). Joint inflammation leads to pain, swelling, and structural changes that limit movement, potentially causing permanent disability, and significantly impacting both dogs' and owners' quality of life (1–3).

Pain management in canine OA typically involves acute and chronic phases. For the acute OA phase, the treatment mainly focuses on reducing pain and inflammation. It is evident that mild symptoms are well managed using analgesics, whereas moderate to severe symptoms can be managed by multimodal combining analgesics such as non-steroidal anti-inflammatory drugs (NSAIDs) or opioids together with local anesthetics, N-methyl-D-aspartate (NMDA) antagonists, or alpha-2 adrenoceptor agonists. For chronic OA pain management, combination therapy is highly recommended (4).

Among analgesics, NSAIDs are the most frequently prescribed medicine for dogs with OA. NSAIDs are usually prescribed for a short duration because of their common gastrointestinal side effects, especially peptic ulcers and renal failure (5–8). Veterinarians usually suggest dog owners use nutraceuticals, such as omega-3 fatty acid, glucosamine, or chondroitin, or non-pharmacological treatment, such as acupuncture and rehabilitation for chronic OA (9, 10). Surgery is another option, although it is quite expensive, and the availability is quite restricted in certain veterinary facilities (4).

Cannabidiol (CBD) has gained increasing popularity in recent years. Previous studies have suggested that CBD may potentially reduce pain, inflammation, anxiety, and epilepsy in dogs (11–14). CBD is believed to offer benefits for dogs with OA, as it is expected to provide pain relief and anti-inflammatory properties. A recent systematic review and meta-analysis of five clinical studies, evaluating the efficacy and safety of CBD in treating canine OA (15), found that using CBD for 4–12 weeks may reduce pain severity scores and pain interference scores. Furthermore, CBD is generally considered safe and well-tolerated in the short term, with few mild adverse events reported, such as vomiting and asymptomatic increase in alkaline phosphatase level (16–20).

The medical use of CBD is recognized among veterinary professionals and dog owners. Two studies investigated the knowledge, perception, and practices of veterinarians in the US (21) and Spain (22), alongside a study on veterinary students in Canada (23). These studies showed that while awareness of CBD's medical potential was widespread among veterinarians and students, a significant knowledge gap concerning its therapeutic application and potential toxicity existed. Most respondents were unable to distinguish between marijuana and its active ingredients; CBD and tetrahydrocannabinol (THC). Participants expressed a shared viewpoint that the existing information on CBD was limited, emphasizing the pressing need for comprehensive clinical research to uncover its efficacy and safety, particularly with respect to long-term use. These studies identified pain, inflammation, anxiety, and seizure management as the most frequently reported known indications for CBD usage. Regional disparities were observed in CBD prescribing practices, as Spanish veterinarians exhibited a notably higher likelihood of prescribing CBD for their patients compared to their American counterparts, with prescription rates of 54.9 and 3.2%, respectively (21–23).

The medical use of CBD in humans is currently well-established in many countries, including Thailand. There are regulations within the industry requiring product registration for human use. Practice guidelines, training, and a certification system for prescribers and dispensers are in place. The Thai government has endorsed and heightened awareness of CBD's medical application (24). The medical applicability of CBD is highly likely to extend to animals. In Thailand, pharmaceutical products intended for veterinary use can be registered as drugs (modern drugs or traditional drugs) with the Thai Food and Drug Administration (25), or especially controlled animal feed with the Department of Livestock Development, Thailand (26). Veterinary drugs are available in veterinary clinics and hospitals and can be dispensed by veterinarians (25). Especially controlled animal feed, including veterinary nutraceuticals, is available in licensed places authorized by the Department of Livestock Development, such as animal food shops, or pet shops, which can be sold without prescription (26).

Currently, there are no CBD products for animals available in Thailand. Given CBD's safety profile and potential efficacy, coupled with a growing inclination toward pet ownership rather than having children, individuals are expected to invest more in enhancing their pets' wellbeing. This trend suggests the potential for CBD products to carve a niche in Thailand's existing market. The objective of this study was to investigate the perspectives of Thai veterinarians regarding CBD products intended for canine OA. The knowledge gained from this research will play a crucial role in developing a strategy to prepare veterinarians to adopt CBD products for canine OA and to prepare a target product profile for CBD products.

## Materials and methods

### Sample and recruitment

Eligible participants were registered Thai veterinarians who met the following criteria: (1) general practitioners or specialists in surgery or rehabilitation, (2) practicing for over 3 years, and (3) having at least one-fourth of their canine patients with OA.

We employed a stratified purposive sampling, aiming to include a minimum of five participants from university hospitals and five from private settings. Potential participants were identified by a researcher (KS) who is a veterinarian in Thailand. Recruitment continued until data saturation was reached, as there were no new emerging ideas in the data collected from the participants (27).

### Interview guide

A semi-structured interview guide was developed to assess the veterinarians' perceptions regarding CBD products for treating canine OA (Table 1). The interview guide was reviewed by two veterinarian experts in treating canine OA in Thailand. This process was done to ensure face validity and clarity of the questions.

**Table 1:** Semi-structured interview guide.

**Part of the interview guide**	**Question items**
Treatment protocol for dogs with OA	1. What are the drug regimens you usually prescribe to dogs with OA? Please explain (drug name, strength, administration, duration of treatment). 2. What are the regimens you usually suggest for maintenance stage of OA? (Please specify nutraceuticals, rehabilitation, other alternative medicines, administrative detail of the suggested regimens, duration of the maintenance) 3. Could you estimate costs of the treatment/episode (both initial treatment and maintenance treatment)?
Perception of CBD products	4. Have you ever heard about the benefits of CBD products for the treatment of animals? What are CBD products indicated for (both diseases and animal species)? 5. Have you ever experienced using CBD products to treat dogs with OA? How long duration? How is your satisfaction with efficacy and/or safety? 6. What are the risks associated with the use of CBD products? Please explain. 7. Have you ever heard that CBD could benefit OA in dogs? Please explain why you believe or do not believe. 8. Have you ever discussed CBD with your colleagues? In what way? 9. Have you ever been asked by pet owners about CBD products? What are they looking for? 10. In your opinion, should a product containing CBD be “drug” or “dietary supplement”? Please explain. 11. In your opinion, should CBD be developed as a product with a single active ingredient or as a combination product? Why? If a combination product is preferred, what other active ingredient(s) should be combined? 12. In your opinion, at what price/month that pet owners can afford the CBD product?
Likeliness of CBD product adoption in Thailand	13. If there is a product containing CBD, would you recommend it to your patients? Why or why not? What condition of OA patients should CBD products be suggested for? 14. Are there any concerns regarding CBD products for your patient? - Safety, efficacy - Special populations e.g., dogs with kidney or liver condition - Issues regarding law and regulation 15. If you can develop CBD products of your own, what would your product be like? - Physical appearance (e.g., crispy snack, meat-like snack, creamy paste) - Flavor
Participant characteristics	16. Gender 17. Practice setting (academia, or private clinic) 18. Specialty (general practitioner, surgeon, rehabilitation) 19. Graduation year

OA, osteoarthritis; CBD, cannabidiol.

### Interview procedure

In-depth interviews were conducted virtually using Zoom or Microsoft Teams applications. Interviews were conducted in the Thai language by trained research assistants (TP, NP, and DL) and lasted ~30 to 60 min. All participants gave verbal consent and were asked for permission to audio-record the interviews.

### Data management and analysis

Interview records were transcribed and coded by the researcher assistants (TP, NP, and DL) and subsequently verified by senior researchers (ON, SV, and KS). Codes from research assistants and researchers were compared to ensure accuracy. If discrepancies in transcribing and coding were raised, they were resolved by the other researchers (CP, PA). Data were analyzed using qualitative content analysis. Quotes from the interviews were translated and presented.

## Results

Twenty potentially eligible participants were initially identified, of whom 11 agreed to participate. Interviews were concluded after the 11^th^ participant as data saturation was achieved. Interviews were conducted during January-March 2023. We presented the findings along with quotes from the interviews. However, it should be noted that the following quotes represent participants' opinions, and they may not necessarily align with scientific evidence.

### Participant characteristics

Participant characteristics are shown in Table 2. Five participants were affiliated with university hospitals, while six were associated with private veterinary clinics. Most participants were male (*n* = 7), possessed specialist qualifications (*n* = 8), including surgery (*n* = 5), rehabilitation (*n* = 2), and internal medicine (*n* = 1), held a Master's or a Doctorate degree (*n* = 7), and practiced in the Bangkok metropolitan area (*n* = 7). A considerable portion of the participants had never prescribed or recommended CBD products to dogs (*n* = 7) and had not received any formal training on CBD (*n* = 7).

**Table 2:** Participant characteristics.

**Characteristics**	**Participants ID**										
	**H01**	**H02**	**H03**	**H04**	**H05**	**P01**	**P02**	**P03**	**P04**	**P05**	**P06**
Gender Male: Female = 7:4	F	M	F	M	F	M	M	M	F	M	M
Highest education in addition to DVM MSc = 5 PhD = 2	PhD	-	PhD	MSc	-	MSc	-	MSc	MSc	MSc	-
Specialty Surgery = 5 Rehabilitation = 2 General practitioner = 3 Internal medicine = 1	Surg	Surg	Surg	Rehab	GP	Rehab	GP	Surg	IM	Surg	GP
Practice year Range 3–27 yr	22	7	27	7	3	32	21	12	13	13	3
Practice setting University hospital = 5 Private clinic = 6	Uni H	Uni H	Uni H	Uni H	Uni H	Priv clinic	Priv clinic	Priv clinic	Priv clinic	Priv clinic	Priv clinic
Practice location Bangkok = 7 Non-Bangkok = 4	Chiang Mai	Song Khla	Chiang Mai	Bangkok	Bangkok	Samut Song kram	Bangkok	Bangkok	Bangkok	Bangkok	Bangkok
% of canine patients diagnosed with osteoarthritis Range 30–100%	100%	30%	>50%	60–80%	30–40%	100%	100%	80%	>50%	60%	60–80%
Have been trained for prescribing cannabidiol products Yes = 4 No = 7	Yes	No	Yes	Yes	No	Yes	No	No	No	No	No
Had experience using cannabidiol products to treat patients Yes = 4 No = 7	No	No	No	Yes	Yes	No	No	Yes	No	No	Yes

### Treatment pattern and costs

Canine patients with OA presented at the clinics varied from large to small breeds. The diversity of OA cases correlated with the historical popularity of different breeds. All veterinarians homogenously stated that treatment of the OA condition could be classified as acute or chronic. During the acute phase, non-steroidal anti-inflammatory drugs (NSAIDs) emerged as a preferred choice for pain relief and anti-inflammation. Firocoxib (*n* = 8) and carprofen (*n* = 7) were the two most frequently prescribed NSAIDs. Supplementary pain medications such as Gabapentin (*n* = 2) and opioid derivatives (*n* = 1) were occasionally co-administered alongside NSAIDs. The duration of NSAIDs prescribed varied from 4–5 days up to 2 months.

Multimodality approaches were commonly reported for chronic OA treatment. Alongside medications, participants mentioned additional treatment options, such as rehabilitation, platelet-rich plasma therapy, shock wave therapy, ultrasound therapy, acupuncture, electrical therapy, or electrical stimulation (ES), including Transcutaneous Electrical Neuromuscular Stimulation (TENS), Neuromuscular Electrical Stimulation (NMES), Electrical Muscle Stimulation (EMS), laser therapy, and stem cell therapy. Ten participants reported the use of nutraceuticals, including extracts from New Zealand green-lipped mussels, omega-3 fatty acids, glucosamine with chondroitin sulfate, and type II collagen. Nutraceuticals were usually recommended for long-term use; however, they put a financial burden on dog owners.

Treatment costs for canine OA were 800–10,000 Thai Baht (THB) per month (~23–285 USD per month), depending on dog owners' willingness and financial capacity. When nutraceuticals no longer demonstrated effectiveness, surgical intervention would be the last treatment resort. One informant, however, rejected the use of nutraceuticals and rehabilitation, asserting that these methods failed to address the root cause of OA.

“*[Product name], a nutraceutical costs 1,200 THB per month. It is considered expensive for many dog owners. Although I recommended the [Product name], it's up to the dog owners”. (H02)*

“*My client told me that nutraceutical is helpful, but he cannot afford to use it continuously. He said he fed his dog every other day”. (P04)*

“*I agree that acute treatment was necessary but disagree with using nutraceuticals and rehabilitation for long-term treatment. This is especially true for OA, which is caused by joint structure abnormality, e.g., joint loosening or dysplasia. Nutraceuticals or rehabilitation did not solve the root cause of the problem; instead, they prolong patients' suffering. Nutraceutical and rehabilitation rather soothe the owners as they perceive they have done something for their beloved pets.” (P02)*

### Awareness and perception of CBD

Participants acknowledged being aware of CBD products; however, half (*n* = 6) were confident and accurately comprehended the difference between CBD and THC, which were the two main active ingredients of *Cannabis sativa*. Also, they were confused about CBD and marijuana. Diverse sources of CBD knowledge were identified: published research papers (*n* = 4), colleagues (*n* = 3), and ongoing local research (*n* = 2).

“*I've heard that CBD can be used in the medical field, but I'm afraid of its addictive effect and worry about its psychological effect.” (H03)*

“*I've heard about it (CBD), but never read about it myself.” (P03)*

Most veterinarians (*n* = 8) indicated that they normally engaged in discussions regarding the benefits and risks of CBD products with their colleagues. All of them reported having a neutral to positive attitude toward CBD products. A few participants mentioned the existence of an online group chat in an application called LINE that was dedicated to discussing the medical use of CBD products.

“*CBD LINE group for veterinarians was established for those interested in the medical use of CBD. The members included academia and practitioners. All members want to see evidence-based information”. (H03)*

### Perceived efficacy and safety of CBD for canine OA

Nine participants reported they believed that CBD can be used for pain relief (*n* = 8), anti-inflammatory (*n* = 5), cancer (*n* = 3), and epilepsy (*n* = 2). Among the eight veterinarians who specified pain relief as an indication of CBD, six were able to explain the mechanism of action of CBD. Notably, participants working in university hospitals were more likely able to provide explanations about the mechanism of CBD compared to those in private veterinary clinics.

Participants were queried about the potential use of CBD for canine OA. Only four participants held a positive attitude regarding the indication of CBD for canine OA.

“*Theoretically, CBD should work well for dogs with OA because cannabinoid receptors distribute centrally and peripherally. But whether the CBD will actually bind with the receptor or not, I'm not sure.” (P06)*

“*CBD, like opioids, suppresses pain signaling in the brain but does not actually reduce pain at the joint. It also has no effect on inflammation.” (H01)*

Participants voiced several concerns related to CBD products, including potential overdose (*n* = 3), hepatotoxicity (*n* = 3), renal toxicity (*n* = 2), addiction (*n* = 2), hypersomnia (*n* = 2), vomiting (*n* = 2), and hypersalivation (*n* = 1).

“*Dosing of CBD is important. If overdosed, CBD could cause internal bleeding in the bone. It could cause mental problems. An organic solvent that is used in the extraction process, if it is not properly eradicated, it could be toxic.” (H01)*

“*From the paper, CBD as an active ingredient does not have serious side effects. What I am concerned about is the quality of CBD products. Side effects could be caused by low-quality CBD products.” (P03)*

“*I am concerned about drug dependence, especially for long-term use. Whether it will have an effect on cognitive function and behavior like other drugs or not.” (P04)*

“*I think dogs could oversleep or vomit from using CBD products.” (P05)*

### Proposed a CBD target product profile

Most veterinarians (*n* = 9) thought that CBD products should be registered as a drug, while a few of them preferred the product to be registered as a nutraceutical (*n* = 1) or either drug or nutraceutical (*n* = 1).

“*CBD products should be classified as ‘drug' because the purposes of the products are to treat diseases and alleviate symptoms. With these purposes, the product falls into the scope of a drug. To get approval for drug status, CBD products must be proved safe and efficacy.” (H01)*

“*Because the information regarding the dose of CBD is important. A dose selection study, together with other non-clinical and clinical studies, should be submitted. Registration of the CBD product as a ‘drug' is more appropriate because the marketing authorization holders are required to provide extensive evidence of pharmacokinetics, pharmacodynamic and clinical studies.” (P06)*

“*Because of the addictive property, CBD product should be under the same control as morphine.” (P02)*

“*The nutraceutical registration requirement is looser than the drug's registration process. It consumes less registration time when compared to drugs. As the nutraceutical, the CBD product will be available for use sooner.” (P01)*

“*Too strict control of CBD may prevent access to a potential product. CBD can be used as a part of multimodal treatment and can be combined with other pain relievers, such as NSAIDs. Thus, if it is a nutraceutical, it would be easier for dog owners to access the product.” (H03)*

Eight veterinarians expressed a preference for CBD as a combination product with other active ingredients. Their rationale for favoring CBD in combination products stemmed from the varying mechanisms of different active ingredients, believed to result in synergistic effects. Veterinarians identified several additional active ingredients that they desired to combine with CBD, including analgesics (*n* = 3), omega-3 fatty acids (*n* = 2), glucosamine (*n* = 1), antioxidants (*n* = 2), and hyaluronic acid (*n* = 1).

“*New CBD products should be developed to meet a multimodal concept. Combining CBD with other active ingredients is considered better than CBD alone as a combination product provides a synergistic effect, uses smaller doses, and has fewer side effects. Moreover, feeding one tablet is more convenient for the dog owner.” (H01)*.

“*I prefer the newly developed CBD product to be a single drug if it has been proven significantly more effective than other available nutraceuticals. If it's not far better than others, a combination may be good.” (P03)*

Concerning the CBD product's dosage form and flavor, participants indicated that the most suitable dosage forms were chewable tablets (*n* = 10), followed by oil (*n* = 5) and soft gelatin capsules (*n* = 2). The flavors frequently recommended included beef (*n* = 5) and liver (*n* = 4), followed by fish, pork, and seafood (*n* = 1 each).

### Potential to prescribe CBD products for canine OA

Intention to prescribe CBD products is crucial, serving as a predictor of actual prescribing behavior. Most veterinarians (*n* = 9) indicated their willingness to prescribe CBD products for canine OA if such products were available in the market. Additionally, seven out of nine veterinarians noted that CBD should not be substituted, but rather integrated with the current treatment. However, their willingness to prescribe CBD hinged on certain conditions: credible efficacy and safety information, a clearly defined dosing regimen, and its prescription for dogs without severe health conditions, possessing normal kidney and liver functions, showing inadequate response to currently available drugs/nutraceuticals, and being within the financial means of the dog owners. Concerns were also voiced regarding the product's quality control, encompassing factors such as the purity of raw materials, consistency of natural substances, and the need to address misconceptions regarding the potentially addictive properties of CBD products.

“*New CBD products would be appropriate for relatively healthy dogs (normal blood chemistry). Late-phase OA dogs may not benefit much from CBD products.” (H02)*

“*CBD products are interesting, but I'm quite concerned about the product quality, especially the consistency of the product from lot to lot, the purity of the CBD, and the efficacy, safety, and quality of CBD products across different brands.” (P03)*

“*I have heard that the CBD extraction process might potentially generate residual substances that could pose a risk to patients. My concerns are related to toxicity and the long-term safety of the CBD product.” (H01)*

“*In my opinion, CBD is not the first resort to treat canine OA. It also cannot replace existing treatment options. Because of the diverse mechanism of action, CBD should be used as a multimodal by combining it with other drugs or nutraceuticals.” (P06)*

## Discussions

This study was conducted as part of the CBD product development. It is the first to explore Thai veterinarians' perceptions of CBD. While all the interviewed veterinarians have heard of CBD and believe it can be used for pain, inflammation, cancer, and epilepsy, the majority still possess insufficient knowledge about CBD, which could mislead the true benefits of CBD. Most have not extensively engaged with scientific papers on the efficacy and safety of CBD and often confuse to differentiate CBD with THC and marijuana. These findings are consistent with prior research by Kogan et al. (21) and Romero et al. (22), which explored veterinarians in the US and Spain. Therefore, comprehensive education is recommended, including continuing education for those in practice and incorporating CBD-related topics into the Schools of Veterinary's curriculum.

The majority of veterinarians expressed a desire for new products aimed at treating chronic OA in dogs, as NSAIDs cannot be used long-term. Previous five clinical studies suggested a trend indicating that CBD could alleviate pain and improve mobility (16–20). However, pain measurement predominantly relied on subjective assessment, and these studies were rated as having a high risk of bias (15). Despite the existing knowledge of CBD's pain relief and anti-inflammatory properties, potentially making it a preferred treatment in canine OA, Thai veterinarians would feel more assured in prescribing CBD if the new product is supported by credible scientific data regarding its safety, effectiveness, and quality. Evidence from well-designed clinical trials as well as meta-analyses of CBD products, are needed.

Thai veterinarians express a preference for CBD products to attain drug status, a classification that not only mandates comprehensive safety and efficacy information but also necessitates a veterinarian's prescription. This designation offers the advantage of enabling healthcare professionals to closely monitor effectiveness and safety. However, the attainment of drug status involves a lengthier dossier preparation process, potentially resulting in delayed access to CBD products. Recognizing the concerns of Thai veterinarians, CBD product developers should formulate an appropriate regulatory strategy to ensure timely approval, along with initial restrictions on distribution, supervised by veterinarians upon launch. Once the efficacy and safety profile of CBD is established among Thai veterinarians, wider product distribution through pet shops and online stores would allow convenient access for pet owners. This approach seeks to strike a balance between the regulation of new products and timely availability, ultimately serving the best interests of all stakeholders; regulators (e.g., Food and Drug Administration), veterinarians, and pet owners.

Unlike Kogan et al. (21) and Romero et al. (22) studies that employed quantitative survey methodologies and engaged hundreds to thousands of participants, this study adopted an in-depth interview approach, involving a smaller yet highly focused group of participants. Despite the limited participant size, all participants included in this study were key opinion leaders or influencers, representing academic and private sectors. Their valuable insights hold substantial potential to effectively inform product development and refine marketing strategies for CBD products. Thus, a quantitative survey study should be further conducted on a larger group of veterinarians in Thailand to investigate the perceptions of CBD for canine OA.

## Conclusions

While the majority of Thai veterinarians are aware of the medical applications of CBD, their familiarity with its efficacy and safety remains limited. Given reliable CBD information, it is likely that Thai veterinarians would be more inclined to recommend CBD products as a multimodality for treating canine OA. During the initial phase, close regulation by veterinarians should be implemented for new CBD products. Once a robust profile of efficacy and safety has been established, these products can then be made accessible and directly managed by pet owners.

## Data availability statement

The original contributions presented in the study are included in the article/supplementary material, further inquiries can be directed to the corresponding author.

## Ethics statement

The studies involving humans were approved by Research Ethics Review Committee for Research Involving Human Research Participants, Chulalongkorn University, Bangkok, Thailand. The studies were conducted in accordance with the local legislation and institutional requirements. The Ethics Committee/institutional review board waived the requirement of written informed consent for participation from the participants or the participants' legal guardians/next of kin because the consent cover letter was sent to the participants with the recruitment email, so they can review it before agreeing to participate. Contact information was available in the consent cover letter and the recruitment information in the event that potential participants have any questions. Verbal consent was obtained at the beginning of the interview. The process of obtaining verbal consent was overseen by the researcher to ensure that all participants are fully informed and that their rights as research subjects are protected.

## Author contributions

ON: Conceptualization, Data curation, Formal analysis, Investigation, Methodology, Project administration, Supervision, Validation, Writing – review & editing. KS: Conceptualization, Data curation, Validation, Writing – review & editing. TP: Data curation, Formal analysis, Investigation, Writing – review & editing. NP: Data curation, Formal analysis, Investigation, Writing – review & editing. DL: Data curation, Formal analysis, Investigation, Writing – review & editing. CP: Conceptualization, Data curation, Formal analysis, Methodology, Validation, Writing – original draft. SV: Formal analysis, Funding acquisition, Validation, Writing – original draft. PA: Conceptualization, Formal analysis, Funding acquisition, Investigation, Methodology, Project administration, Supervision, Validation, Writing – original draft, Writing – review & editing.
